# Antimicrobial Effect of *Lippia sidoides* and Thymol on *Enterococcus faecalis* Biofilm of the Bacterium Isolated from Root Canals

**DOI:** 10.1155/2014/471580

**Published:** 2014-02-06

**Authors:** H. N. H. Veras, F. F. G. Rodrigues, M. A. Botelho, I. R. A. Menezes, H. D. M. Coutinho, J. G. M. da Costa

**Affiliations:** ^1^Laboratory of Natural Products Research (LPPN), Regional University of Cariri, Crato, CE, Brazil; ^2^Northeast Biotechnology Network, Brazil; ^3^Laboratory of Pharmacology and Molecular Chemistry (LFQM), Regional University of Cariri, Crato, CE, Brazil; ^4^Laboratory of Microbiology and Molecular Biology (LMBM), Department of Biological Chemistry, Regional University of Cariri, Cel. Antonio Luis Street, 1161 Pimenta, 63105-000 Crato, CE, Brazil

## Abstract

The species *Lippia sidoides* Cham. (Verbenaceae) is utilized in popular medicine as a local antiseptic on the skin and mucosal tissues. *Enterococcus faecalis* is the bacterium isolated from root canals of teeth with persistent periapical lesions and has the ability to form biofilm, where it is responsible for the failure of endodontic treatments. Essential oil of *L. sidoides* (EOLS) and its major component, thymol, were evaluated for reducing the CFU in biofilms of *E. faecalis in vitro*. The essential oil was obtained by hydrodistillation and examined with respect to the chemical composition, by gas chromatography-mass spectrometry (GC-MS). The GC-MS analysis has led to the identification of thymol (84.9%) and p-cymene (5.33%). EOLS and thymol reduced CFU in biofilms of *E. faecalis in vitro* (time of maturation, 72 h), with an exposure time of 30 and 60 min at concentrations of 2.5 and 10%. There was no statistical difference in effect between EOLS and thymol, demonstrating that this phenolic monoterpene was the possible compound responsible for the antimicrobial activity of EOLS. This study provides a basis for the possible utilization of EOLS as an adjuvant in the treatment of root canals that show colonization by *E. faecalis*.

## 1. Introduction

Advances in technology dealing with the problematics of the emergence of microorganisms increasingly more resistant to conventional antimicrobials have prompted the search for new substances of natural origin, with greater or equal efficacy as those drugs usually used [1]. One of the greatest problems faced is the formation of biofilm by bacteria. Organisms living in communities as in biofilms can tolerate changes in pH and the action of oxygen radicals, disinfectants, and antibiotics better than cells living in a planktonic manner [2].

*Enterococcus faecalis *is a Gram-positive coccus which generally occurs as pairs and short chains, and it is catalase negative [3]. It is the most common and, occasionally, single bacterium most often isolated from root canals of teeth with persistent periapical lesions [4]. The ability to form biofilm by this genus allows the colonization of inert and biological surfaces, protection against antimicrobial agents and the action of phagocytes, mediating adhesion, and invasion of host cells [5]. Therefore, the formation of biofilm is responsible for the failure of endodontic treatments [6].

Therefore, studies are conducted in order to determine the efficacy of natural products in the control of biofilm. The majority of these studies investigate the control of dental biofilm (bacterial plaque), such as the gel and extract of *Punica granatum *(pomegranate) [7, 8], *Lippia sidoides *(alecrim pimenta) [9], *Coffea arabica *(coffee) [10], and *Rheedia brasiliensis *(bacupari) [11].

*Lippia *is the second largest genus of the family Verbenaceae and includes many medicinal and aromatic species, which are found in South America (approximately 70–75% of the known species are in Brazil), Central America, and tropical Africa [12]. *Lippia sidoides *Cham., popularly known as “alecrim pimenta,” is a shrub native to the semiarid region of Northeast Brazil. It is widely employed in popular medicine as a local antiseptic on skin and mucosal tissues. The therapeutic effect of *L. sidoides *is attributed mainly to the presence of thymol, a substance with high antimicrobial activity, which is the major component in the plant's essential oil and is also found in hydroalcoholic extracts of the plant [13]. This study investigated the *in vitro *antimicrobial activity of the essential oil of *Lippia sidoides *and of its major component, thymol, on biofilm of *Enterococcus faecalis*.

## 2. Material and Methods

### 2.1. Plant Material

Leaves of *Lippia sidoides *Cham. were collected in August 2010, from the Small Aromatic and Medicinal Plants Garden of the Natural Products Research Laboratory (LPPN) at Regional University of Cariri (URCA), Crato County, Ceara State, Brazil. A voucher specimen was deposited in the Herbarium Caririense Dardano of Andrade Lima of the Department of Biological Sciences (URCA) under registration number 3038.

### 2.2. Drug

Thymol was obtained from Sigma Chemical Corporation, St. Louis, MO, USA.

### 2.3. Essential Oil Isolation

Samples of *L. sidoides *fresh leaves (140 g) were triturated and submitted to hydrodistillation process, in a Clevenger-type apparatus for 2 h. The collected essential oil was subsequently dried with anhydrous sodium sulfate (Na_2_SO_4_) and stored refrigerated at <10°C until analyzed and tested.

### 2.4. Analysis of the Essential Oil

Analysis by CG/MS of the essential oil was carried out on a Hewlett-Packard Model 5971 GC/MS using a nonpolar DB-1 fused silica capillary column (30 m × 0.25 mm i.d., 0.25 m film thickness). Helium was the carrier gas, and flow rate was 0.8 mL/min, using split mode. The injector temperature and detector temperature were 250°C and 200°C, respectively. The column temperature was programmed from 35°C to 180°C at 4°C/min and then from 180°C to 250°C at 10°C/min. Mass spectra were recorded from 30 to 450 m/z. Individual components were identified by matching their 70 eV mass spectra with those of the spectrometer database using the Wiley L-built library and two other MS library searches using retention indices as a preselection routine [14], as well as by visual comparison of the fragmentation pattern with those reported in the literature [15].

### 2.5. Evaluation of the Inhibition of Biofilm Formation

A pure culture of *Enterococcus faecalis *ATCC 4083 was subcultured on a BHI agar plate for 24 h at 35 ± 2°C under aerobic conditions. After growth, isolated colonies were suspended in tubes containing 5 mL of BHI broth. After mixing, the suspension was adjusted to a concentration equivalent to 6.0 on the McFarland scale. Nitrocellulose membrane filters (0.22 *μ*m porosity, 13 mm in diameter) were placed on the BHI agar plates, and then 50 *μ*L of the bacterial suspension was placed on each membrane. The plates were incubated for 72 h in air at 35 ± 2°C. The essential oil of *Lippia sidoides *(EOLS) and thymol were dissolved separately in DMSO and were then diluted with sterile distilled water at concentrations of 2.5% and 10%. Sodium hypochlorite was used as the positive control and DMSO as the negative control, both at the same concentrations as the samples analyzed. After the incubation period, the biofilms were immersed in 3 mL of each solution, at different concentrations, for 30 and 60 min. After the exposure time, the membranes were carefully transferred to 3 mL of neutralization broth D/E (for EOLS, thymol, and DMSO) or to 3 mL of 1% sodium thiosulfate (for sodium hypochlorite) to stop the possible antimicrobial action of the test agent. Next, the membranes were vortexed for 30 s to resuspend the microorganisms [16, 17]. Finally, the suspensions were diluted 10 times for counting of colony forming units (CFU/mL) utilizing D/E agar, in triplicate [18].

### 2.6. Statistical Analysis

The results were expressed as means ± standard error of mean (S.E.M.) and statistical significance was determined by two-way ANOVA followed by Bonferroni's test, with the level of significance set at *P* < 0.05 using the program *GraphPadPrism 5.0.*

## 3. Results

**Figure 1:**
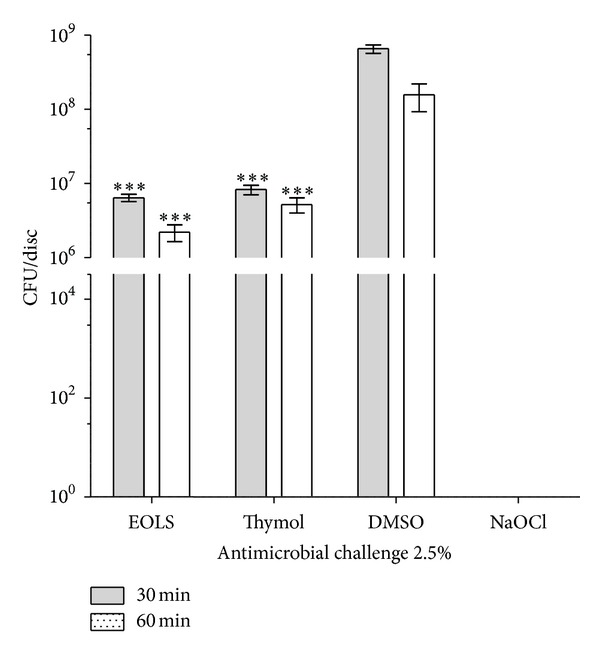
Susceptibility of biofilms of *Enterococcus faecalis *to antimicrobial challenge at 2.5% (v/v) for 30 or 60 min exposure times. EOLS, essential oil of *Lippia sidoides*; DMSO, dimethyl sulfoxide (negative control); and NaOCl, sodium hypochlorite (positive control). The vertical bars indicate the standard deviation (*n* = 3). ***P < 0.001  compared with DMSO (two-way ANOVA followed by the Bonferroni test).

**Figure 2:**
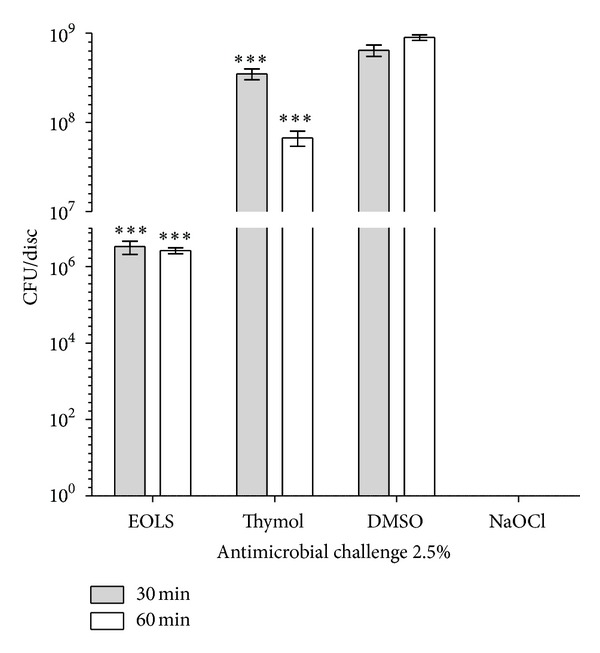
Susceptibility of biofilms of *Enterococcus faecalis *to antimicrobial challenge at 10.0% (v/v) for 30 or 60 min exposure times. EOLS, essential oil of *Lippia sidoides*; DMSO, dimethylsulfoxide (negative control); and NaOCl, sodium hypochlorite (positive control). The vertical bars indicate the standard deviation (*n* = 3). ***P < 0.001 compared with DMSO (two-way ANOVA followed by the Bonferroni test).

The essential oil obtained by hydrodistillation of fresh leaves of *L. sidoides *gave a yield of 1.06% (w/w). The major constituents of the essential oil of *L. sidoides *were thymol (84.9%), *p*-cymene (5.33%), and ethyl methyl carvacrol (3.01%) (see Table 1). The means of colony forming units (CFU) per disk of *E. faecalis *biofilm after the exposure time (30 or 60 min) with 2.5% and 10% solutions of EOLS, thymol, DMSO, and sodium hypochlorite (NaOCl) are shown in Figures 1 and 2, respectively. NaOCl was the most effective antimicrobial agent, eliminating 99.99% of the bacteria with the concentrations and exposure times utilized in this study.

Figure 1 shows that 2.5% DMSO, the negative control, had no significant effect on cell viability for both times tested, resulting in 6.5 × 10^8^ and 1.5 × 10^8^ CFU in 30 and 60 min of exposure. After exposure to EOLS for 30 and 60 min, CFU count in relation to DMSO control was significantly reduced (*P* < 0.001) to 6.4 × 10^6^ and 2.2 × 10^6^ CFU. Thymol decreased significantly (*P* < 0.001) the CFU count to 8.3 × 10^6^ and 5.2 × 10^6^, respectively. There were no statistical differences (*P* > 0.05) between EOLS and thymol effects for the designated exposure times.

After 30 and 60 min of exposure, 10% DMSO had no significant effect on cell viability, resulting in 6.4 × 10^8^ and 9.0 × 10^8^ CFU, respectively. CFU counts for biofilms exposed to EOLS and thymol at 10% in relation to the negative control were significantly reduced (*P* < 0.001) to 3.3 × 10^6^ and 2.6 × 10^6^ and 3.5 × 10^8^ and 6.7 × 10^7^ CFU, respectively. There was a statistical difference (*P* < 0.001) in mean CFU counts between EOLS and thymol for 30 min exposure. On the other hand, exposure of biofilms to EOLS and thymol for 60 min showed no difference (*P* > 0.05) (Figure 2).

## 4. Discussion

In some studies, the level of thymol present in the essential oil of the leaves can vary from 34.2 to 95.1% [19, 20]. This variation in level of constituents in essential oil can be influenced by the cultivation and development conditions (type of soil and climate), harvest and postharvest processing (time of day and season) [21] (Gil et al. 2002). The majority of microorganisms do not exist as a culture of free-living cells, but rather associated with a living or inert surface, forming a structured community of cells surrounded by a polysaccharide matrix [22] (Costerton et al. 1999). There are various *in vitro *methods that are used to evaluate the effectiveness of antimicrobial agents against biofilms, but the results are conflicting in works utilizing the same test substances and the same microorganisms but different methods [16, 17, 23, 24]. The protocols utilized in this study were adapted from Abdullah et al. and Enright et al. studies This method is feasible and rapid, besides allowing the comparison of various antimicrobial challenges against microorganisms present in biofilm [16, 17].

The virulence of *E. faecalis *in root canals can be related to its capacity to resist intracanal drug treatment and to its ability to survive in the root canal as the only microorganism without the support of other bacteria, forming biofilms [25]. The irrigation of root canals is an important step in disinfection and is an integral part of procedures of endodontic treatment. Currently, the irrigant most often used is sodium hypochlorite (NaOCl) due its strong antimicrobial activity, but the main disadvantage of its use in dental treatment is its toxicity to patient's tissues [26].

Structured bacteria in biofilm behave differently when exposed to chemical substances, because polymeric substances that make up the biofilm matrix hamper the diffusion of chemical substances and antibiotics [27, 28]. The susceptibility of biofilm is directly related to time of exposure and to the concentration of the substance, besides the phase of biofilm development [17]. The speed of penetration of the substance varies according to the microorganism and composition of exopolysaccharide matrix [22]. Therefore, our results demonstrate that EOLS and thymol are capable of reducing *E. faecalis *CFU in biofilms *in vitro *(time of maturation, 72 h) with an exposure time of 30 and 60 min, at concentrations of 2.5 and 10%. At 2.5%, there were no statistical differences (*P* > 0.05) between exposure time and the samples tested, where thymol was responsible for the antimicrobial activity of EOLS against the biofilm. On the other hand, the higher concentration of thymol (10%) was not as effective as the lower concentration (2.5%), which was not the case for EOLS, showing the same activity at both concentrations and with both exposure times. This is the first report on the action of EOLS against biofilms of *E. faecalis*.

The mechanisms by which EOLS and thymol kill microorganisms present in biofilms are still not well elucidated. However, studies of the mechanism of action of carvacrol and thymol on biofilms remain unclear; their amphipathic nature could account for the observed effects. The relative hydrophilicity of carvacrol and thymol may allow their diffusion through the polar polysaccharide matrix, whilst the prevalent hydrophobic properties of these compounds could lead to specific interactions with the bacterial membrane causing the dispersion of the polypeptide chains of the cell membrane and destabilizing the cell [29–31]. This hypothesis is supported by the electron micrographs of damaged cells and the significant increase of the cell constituents' release demonstrated that thymol and other essential oil combinations affected the cell membrane integrity [32].

A preparation based on essential oils of *Eucalyptus globulus*, *Melaleuca alternifolia*, *Thymus *sp., and *Syzygium aromaticum*, containing mainly monoterpenes, demonstrated, *in vitro*, reduced adherence of *Staphylococcus epidermidis *and formation of biofilm [33]. The combination of thymol and chlorhexidine gluconate demonstrated synergistic activity against *S. epidermidis *biofilm [34]. Braga et al. found that thymol also interferes with the adherence of *C. albicans *on mucosal cells, and they suggested that this compound can significantly interfere not only with the initial phases of biofilm formation but also with its maturation, since it effectively inhibits the metabolic activity of biofilm.

According to Nostro et al., thymol is as much hydrophilic as hydrophobic, which can favor the diffusion of this compound through the polysaccharide layer of biofilm and reach the bacterial cells to exert its antimicrobial effect by altering membrane permeability [31]. This hypothesis is supported by the results obtained in various clinical studies with mouthwashes or toothpastes containing EOLS, which have demonstrated a decrease in bacterial plaque [35, 36].

Therefore, our results provide a basis for the possible utilization of EOLS or its major component, thymol, as adjuvants in the treatment of root canals that show colonization by *E. faecalis*. However, preclinical studies are necessary to evaluate the true efficacy of these products and the concentration needed to kill biofilm bacteria *in vivo*.

## Figures and Tables

**Table 1:** Chemical components of *Lippia sidoides* fresh leaves essential oil.

Compounds	Tr (min)	IK*	(%)
*p-*cymene	4.2	1020	5.33
1,8-Cineol	4.4	1031	1.68
*γ-*Terpinene	5.0	1060	1.32
Ether ethyl carvacrol	9.7	1164	3.01
Thymol	11.8	1288	84.9
Carvacrol	12.9	1292	0.41
*β-*Caryophyllene	15.1	1418	1.17

Total			97.82

*Relative retention indices experimental: n-alkanes were used as reference points in the calculation of relative retention indices.

## References

[1] Brotz-Oesterhelt H, Beyer D, Kroll HP (2005). Dysregulation of bacterial proteolytic machinery by a new class of antibiotics. *Natural Medicines*.

[2] Jefferson KK (2004). What drives bacteria to produce a biofilm?. *FEMS Microbiology Letters*.

[3] Teixeira LM, Facklam RR (2005). Enterococcus. *Topley and Wilson's Microbiology and Microbial Infections*.

[4] Rôças IN, Siqueira JF, Santos KRN (2004). Association of Enterococcus faecalis with different forms of periradicular diseases. *Journal of Endodontics*.

[5] Baldassarri L, Creti R, Montanaro L, Orefici G, Arciola CR (2005). Pathogenesis of implant infections by enterococci. *International Journal of Artificial Organs*.

[6] Lin LM, Skribner JE, Gaengler P (1992). Factors associated with endodontic treatment failures. *Journal of Endodontics*.

[7] Alsaimary IE (2010). Efficacy of some antibacterial agents against Streptococcus mutans associated with tooth decay. *The Internet Journal of Microbiology*.

[8] Salgado ADY, Maia JL, Pereira SLDS, de Lemos TLG, Mota OMDL (2006). Antiplaque and antigingivitis effects of a gel containing Punica granatum Linn extract. A double-blind clinical study in humans. *Journal of Applied Oral Science*.

[9] Botelho MA, dos Santos RA, Martins JG (2009). Comparative effect of an essential oil mouthrinse on plaque, gingivitis and salivary Streptococcus mutans levels: a double blind randomized study. *Phytotherapy Research*.

[10] Landucci LF, Oliveira LD, Brandão EHS, Koga-Ito CY, Jardim Júnior EG, Jorge AOC (2010). Efeitos de Coffea arabica sobre a aderência de Streptococcus mutans à superfície de vidro. *Brazilian Dental Science*.

[11] Almeida LSB, Murata RM, Yatsuda R (2008). Antimicrobial activity of Rheedia brasiliensis and 7-epiclusianone against Streptococcus mutans. *Phytomedicine*.

[12] Viccini LF, Pierre PMO, Praça MM (2005). Chromosome numbers in the genus Lippia (Verbenaceae). *Plant Systematics and Evolution*.

[13] Matos F, Oliveira F (1998). Lippia sidoides Cham.: farmacognosia, química e farmacologia. *Revista Brasileira de Farmácia*.

[14] Alencar J, Craveiro A, Matos F, Machado M (1990). Kovats indices simulation in essential oils analysis. *Química Nova*.

[15] Adams R (2001). *Identification of Essential Oil Components by Gas*.

[16] Abdullah M, Ng Y-L, Gulabivala K, Moles DR, Spratt DA (2005). Susceptibilties of two Enterococcus faecalis phenotypes to root canal medications. *Journal of Endodontics*.

[17] Enright MC, Robinson DA, Randle G, Feil EJ, Grundmann H, Spratt BG (2002). The evolutionary history of methicillin-resistant Staphylococcus aureus (MRSA). *Proceedings of the National Academy of Sciences of the United States of America*.

[18] Dey BP, Engley FB (1983). Methodology for recovery of chemically treated Staphylococcus aureus with neutralizing medium. *Applied and Environmental Microbiology*.

[19] Girão VCC, Nunes-Pinheiro DCS, Morais SM, Sequeira JL, Gioso MA (2003). A clinical trial of the effect of a mouth-rinse prepared with Lippia sidoides Cham essential oil in dogs with mild gingival disease. *Preventive Veterinary Medicine*.

[20] Fontenelle ROS, Morais SM, Brito EHS (2007). Chemical composition, toxicological aspects and antifungal activity of essential oil from Lippia sidoides Cham. *Journal of Antimicrobial Chemotherapy*.

[21] Gil A, de la Fuente EB, Lenardis AE (2002). Coriander essential oil composition from two genotypes grown in different environmental conditions. *Journal of Agricultural and Food Chemistry*.

[22] Costerton JW, Stewart PS, Greenberg EP (1999). Bacterial biofilms: a common cause of persistent infections. *Science*.

[23] Seabra EJG, Lima IPC, Barbosa SV, Lima KC (2005). Atividade antimicrobiana “in vitro” de compostos a base de hidróxido de cálcio e tergentol em diferentes concentrações sobre bactérias orais. *Acta Cirurgica Brasileira*.

[24] Arias-Moliz MT, Ferrer-Luque CM, Espigares-García M, Baca P (2009). Enterococcus faecalis biofilms eradication by root canal irrigants. *Journal of Endodontics*.

[25] Paradella TC, Koga-Ito CY, Jorge AOC (2007). Enterococcus faecalis: considerações clínicas e microbiológicas. *Revista de Odontologia da UNESP*.

[26] Bowden JR, Ethunandan M, Brennan PA (2006). Life-threatening airway obstruction secondary to hypochlorite extrusion during root canal treatment. *Oral Surgery, Oral Medicine, Oral Pathology, Oral Radiology and Endodontology*.

[27] Stoodley P, Sauer K, Davies DG, Costerton JW (2002). Biofilms as complex differentiated communities. *Annual Review of Microbiology*.

[28] Xavier J, Picioreanu C, Almeida J, Loosdrecht M (2003). Monitorização e modelação da estrutura de biofilmes. *Boletim de Biotecnologia*.

[29] Cowan MM (1999). Plant products as antimicrobial agents. *Clinical Microbiology Reviews*.

[30] Soumya EA, Saad IK, Hassan L, Ghizlane Z, Hind M, Adnane R (2011). Carvacrol and thymol components inhibiting Pseudomonas aeruginosa adherence and biofilm formation. *African Journal of Microbiology Research*.

[31] Nostro A, Blanco AR, Cannatelli MA (2004). Susceptibility of methicillin-resistant staphylococci to oregano essential oil, carvacrol and thymol. *FEMS Microbiology Letters*.

[32] Lv F, Liang H, Yuan Q, Li C (2011). In vitro antimicrobial effects and mechanism of action of selected plant essential oil combinations against four food-related microorganisms. *Food Research International*.

[33] Al-Shuneigat J, Cox S, Markham J (2005). Effects of a topical essential oil-containing formulation on biofilm-forming coagulase-negative staphylococci. *Letters in Applied Microbiology*.

[34] Karpanen TJ, Worthington T, Hendry ER, Conway BR, Lambert PA (2008). Antimicrobial efficacy of chlorhexidine digluconate alone and in combination with eucalyptus oil, tea tree oil and thymol against planktonic and biofilm cultures of Staphylococcus epidermidis. *Journal of Antimicrobial Chemotherapy*.

[35] Nunes RS, Lira AAM, Lacerda CM, da Silva DOB, da Silva JA, de Santana DP (2006). Obtenção e avaliação clínica de dentifrícios à base do extrato hidroalcoólico da Lippia sidoides Cham (Verbenaceae) sobre o biofilme dentário. *Revista de Odontologia da UNESP*.

[36] Lobo PLD, Fonteles CSR, de Carvalho CBM (2011). Dose-response evaluation of a novel essential oil against Mutans streptococci in vivo. *Phytomedicine*.

